# A multicentre, randomised controlled trial to compare the clinical and cost-effectiveness of Lee Silverman Voice Treatment versus standard NHS Speech and Language Therapy versus control in Parkinson’s disease: a study protocol for a randomised controlled trial

**DOI:** 10.1186/s13063-020-04354-7

**Published:** 2020-05-27

**Authors:** C. M. Sackley, C. Rick, P. Au, M. C. Brady, G. Beaton, C. Burton, M. Caulfield, S. Dickson, F. Dowling, M. Hughes, N. Ives, S. Jowett, P. Masterson-Algar, A. Nicoll, S. Patel, C. H. Smith, R. Woolley, C. E. Clarke, on behalf of the PD COMM Collaborative Group, A. Church, A. Davey, C. Gallagher, A. Conroy, S. Bailey, B. Done, D. Davies, S. Sveinbjornsdottir, M. Kasti, K. Allen, J. Colnet, J. Riches, L. Kittridge, L. Morris, C. Waszkiewicz, V. Lyell, V. Page, N. Bassford, H. Rayner, E. Henderson, S. Abraham, J. V. Hindle, S. Jones, P. Martin-Forbes, C. Watkins, A. Roberts, E. Newcombe, L. Bibby, L. Matthews, J. Roberts, S. Thomas, H. Hawthorne, K. Clewley, S. Lord, J. Roberts, G. Bretag, S. Noble, D. McGhee, H. Mcclure, A. Ling Zhi Teo, R. Wheeldon, C. Wilkinson, M. Oprea, G. Van Duyvenvoorde, N. Wilson, L. Evans, R. Belshaw, A. Clarke, M. Turner, C. Thompson, R. Saha, J. Aram, D. Mullan, J. Newman, K. Micabel, H. Robinson, K. Chick, J. Gaylard, J. Cochrane, E. C. Thomas, A. Kissick, P. Wright, B. Mohamed, S. Mahon, T. Williams, S. Appleton, N. Elliott, L. Evans, J. Ridley, A. Funaki, P. Daly, J. Hackworth, K. Timms, L. Evison, A. Bajracharya, M. Silverdale, K. Andrews, A. Davies, R. Bedford, A. Jones, D. Mournfield, L. Howieson, V. Price, J. Hunter, A. Baggs, L. Evans, R. Norton, M. Holland, K. Pointon, S. Kulkarni, S. Bobeldijk, S. Beames, J. Cavanagh, S. Sudlow, G. Lennox, R. Whittaker, D. Nelson, S. Pegler, C. Drayson, P. Turner, J. Stockwell, T. Andrews, E. White, E. Turner, T. Burnay, C. Hickey, S. Warner, R. Buccoliero, C. Isles, C. Stemp, J. Guy, C. Bennett, S. Smith, P. Randall, L. Ware, J. Cann, L. Sautin, S. Grobler, S. Adjei, A. Hankin, D. Ovayolu, M. Dobbs, O. Joyce, R. Humphreys, A. Jha, C. Holbrook, K. Rowsell, F. Johnson, H. Thornley, S. Conway, C. James, S. Murrow, M. Hughes, K. Pope, C. MacPhee, E. Williams, R. Hughes, A. Evans, A. Richmond, K. Pilborough, K. Campbell, S. E. Davies, A. Taylor, S. Thomas, D. Asandei, T. Majeed, J. Dawber, S. Furey, A. Oppetit, J. Birt, M. Hare, V. Fleming, A. Timoroksa, H. Al-Nufoury, S. Sharp, L. Freimann, I. Mavroudis, J. Alty, E. Richfield, J. Harrison, V. Smith, T. Joyce, J. Bamford, S. Jamieson, J. Cosgrove, S. Butterworth, E. Sacre, L. Makawa, P. Duggan-Carter, C. Arnold, K. Brown, P. Mpofu, C. Joyce, S. Henderson, C. Wiseman, L. Hyne, B. Stevens, A. Wood, D. Holland, V. Smith, J. Juada, S. Molloy, C. Pavel, M. Dhanarante, T. Adedoyin, C. Rowbottom, L. Little, R. Choudhury, L. Prados, A. Kelly, R. Eggers, T. Saifee, P. Poku, L. Niepage, D. Ahearn, A. Fountain, A. Curran, A. Watt, M. Wilson, A. Anderson, M. Graham, J. Taylor, P. Hewat, S. Donaldson, H. Moores-Poole, C. Angus, S. Coull, R. Davy, A. Gilmour-Graham, T. Mcilroy, A. Kendall, S. Pal, E. Messeder, D. Thomson, V. Johnston, P. Raby, S. Kinnear, P. F. Smith, S. Wishart, D. Grosset, J. Burns, A. L. Cunnington, E. Newman, C. Vennard, C. Dalton, T. Murphy, G. Ralph, A. Ritchie, C. Nelson, A. McEntee, P. Fowley, H. Hare, G. Beaton, M. Wilson, D. McDonald, J. Finlayson, A. Donaldson, S. Sutherland, S. Bramley, C. Dunn, M. Wallis, S. Hewitt, H. Morgan, A. Falconer, L. Peacock, A. McAlpine, C. McBrearty, A. Lowe, N. Findlay, A. Adam, C. Tearney, J. Picken, K. MacKenzie, L. McCallum, L. Smith, M. Sidney, P. Downie, L. Donnelly, R. McAllister, S. Campbell, S. Maclachlan, L. Shearer, K. Campbell, G. Duncan, S. Marrinan, M. Dewar, J. Kerr, L. Killin, A. Peters, A. Stewart, T. Daniels, A. Darbyshire, I. McCoy, U. Duff, F. Young, S. Orr, C. Telford, D. Fraser, S. Borthwick, H. Bailey, L. Karbownicki, E. McLeod, D. Sutherland, E. Sammler, L. Whyte, C. Young, L. Gillies, L. Gall, J. Dallas, L. Cassidy, E. Letham, V. Salisbury, L. Anderson, C. Hutton, S. Waggett, D. Anderson, A. Mcgee, S. Cooper, G. Mamutse, A. Niruban, A. Bath, A. Wiltshire, M. Harmer, C. Wright, J. Graham, K. Richardson, J. Tyler, L. Isaacs, N. Crow, S. Pinnell, W. Neale, L. Maloney, R. Weller, K. Young, C. Squire, A. Whone, Y. Hernandez, H. Findlay, K. King, L. Gethin, S. Ticehurst, A. Swift, J. Short, J. Dean, C. Westcott, K. Thomas, S. Cottrell, D. Kruszynska, S. Kamath, Q. Ma, J. Hall, R. Wilson, H. Goodhand, K. Mellows, L. J. Cottam, T. Behan, J. Gibson, E. Lomas, J. Kirk, L. Smith, J. Benson, J. Raw, P. Mulligan, A. Ansari, R. Irving, A. Javed, S. Hussain, L. Johnson, R. Joseph, J. Brooke, J. Melville, M. McCormack, J. Stockley, D. Ganderton, A. Cherriman, J. Price, C. Douglas, C. Cooter, J. Bushell, R. Sheridan, C. Browning, K. Polverino, T. Malone, S. Jackson, A. Foden, R. James, S. Hayes, L. Roberts, E. Davis, C. Clarke, D. Nicholl, A. Majeed, M. T. Oo, K. Blachford, A. Boughey, J. Kaur, S. Kaur, M. Awan, S. Rahman, J. Round, D. Gandecha, S. Williams, S. Dealing, H. Moss, L. Talbot, S. Cooper, R. Sophia, J. Allen, S. Cox, C. Moreira, D. Woolven, D. Sharratt, E. Foster, H. Hurren, J. Watson, S. Northover, D. Green, A. Treloggen, C. Pawley, K. Beesley, K. Milne, L. Howard, S. Craw, A. Lewis, A. Whitcher, C. Vickers, T. Russell, A. Sykes, H. Meikle, N. Loraine, M. Steiger, H. Treloar, L. Roebuck, M. Taylor, R. Nashed, J. Garfield-Smith, S. Mills, H. Griffin, C. Marshall, G. De Selincourt, V. Queen, M. Stone, M. Farrow-Jones, E. Sturdy, K. Almedilla, F. Fitzsimmons, M. Alison, F. Rogers, B. Reed, M. Pinkney, S. Jones, S. Muzerengi, M. Johnson, S. Stafford, E. Parmar, J. Albutt, S. Kaur, M. Awan, S. Rahman, K. Leahy, T. Allain, M. Sritharan, A. Daniell, K. Kunsteinaite, S. Slade, F. Pimbblet, C. Killourhey, E. Wales, C. Hughes, G. Horsfield, L. Mercer, Z. Roberts, K. Stock, M. Evans, S. Boyd, L. King, J. Birch, S. Anderson, C. Evans, N. Stapleton, U. Magennis, R. Vernall

**Affiliations:** 1 Population Health Sciences, Addison House, King’s College London, Guy’s Campus, London, SE1 1UL UK; 2 School of Health Science, University of Nottingham, QMC, Nottingham, NG7 2HA UK; 3 Nottingham Clinical Trials Unit, University of Nottingham, Building 42, University Park, Nottingham, NG7 2RD UK; 4 Birmingham Clinical Trials Unit, University of Birmingham, Birmingham, B15 2TT UK; 5 NMAHP Research Unit, Glasgow Caledonian University, Glasgow, G4 0BA UK; 6 Queen Elizabeth Hospital, NHS Greater Glasgow and Clyde, Glasgow, UK; 7 School of Allied and Public Health Professions, Canterbury Christ church University, Canterbury, CT1 1QU UK; 8 Bangor Institute for Health and Medical Research, School of Healthcare Sciences, Bangor University, Bangor, UK; 9 Cambridge Clinical Trials Unit, Cambridge University Hospitals NHS Foundation Trust, Cambridge, CB2 0QQ UK; 10 Health Economics, University of Birmingham, Birmingham,, B15 2TT UK; 11 Division of Psychology and Language Science, Faculty of Brain Sciences, University College London, London, UK; 12 Institute for Applied Health Research, University of Birmingham, Birmingham, B15 2TT UK; 13 Department of Neurology, Sandwell and West Birmingham Hospitals NHS Trust, Birmingham,, B18 7QH UK

**Keywords:** Idiopathic Parkinson’s disease, Lee Silverman voice treatment, Speech and language therapy, Randomised controlled trial, Dysarthria

## Abstract

**Background:**

Parkinson’s disease (PD) affects approximately 145,519 people in the UK. Speech impairments are common with a reported prevalence of 68%, which increase physical and mental demands during conversation, reliance on family and/or carers, and the likelihood of social withdrawal reducing quality of life. In the UK, two approaches to Speech and Language Therapy (SLT) intervention are commonly available: National Health Service (NHS) SLT or Lee Silverman Voice Treatment (LSVT LOUD®). NHS SLT is tailored to the individuals’ needs per local practice typically consisting of six to eight weekly sessions; LSVT LOUD® comprises 16 sessions of individual treatment with home-based practice over 4 weeks. The evidence-base for their effectiveness is inconclusive.

**Methods/design:**

PD COMM is a phase III, multicentre, three-arm, unblinded, randomised controlled trial. Five hundred and forty-six people with idiopathic PD, reporting speech or voice problems will be enrolled. We will exclude those with a diagnosis of dementia, laryngeal pathology or those who have received SLT for speech problems in the previous 2 years. Following informed consent and completion of baseline assessments, participants will be randomised in a 1:1:1 ratio to no-intervention control, NHS SLT or LSVT LOUD® via a central computer-generated programme, using a minimisation procedure with a random element, to ensure allocation concealment. Participants randomised to the intervention groups will start treatment within 4 (NHS SLT) or 7 (LSVT LOUD®) weeks of randomisation.

Primary outcome: Voice Handicap Index (VHI) total score at 3 months. Secondary outcomes include: VHI subscales, Parkinson’s Disease Questionnaire-39; Questionnaire on Acquired Speech Disorders; EuroQol-5D-5 L; ICECAP-O; resource utilisation; adverse events and carer quality of life. Mixed-methods process and health economic evaluations will take place alongside the trial. Assessments will be completed before randomisation and at 3, 6 and 12 months after randomisation.

The trial started in December 2015 and will run for 77 months. Recruitment will take place in approximately 42 sites around the UK.

**Discussion:**

The trial will test the hypothesis that SLT is effective for the treatment of speech or voice problems in people with PD compared to no SLT. It will further test whether NHS SLT or LSVT LOUD® provide greater benefit and determine the cost-effectiveness of both interventions.

**Trial registration:**

International Standard Randomised Controlled Trials Number (ISRCTN) Registry, ID: 12421382. Registered on 18 April 2016.

## Background

In prevalence and years lived with disability, Parkinson’s disease (PD) is the fastest-growing neurological disorder in the world [1], and it affects approximately 145,519 people in the UK. PD is a complex, progressive condition with a range of motor and non-motor symptoms [2]. Speech impairments are common in the PD population with a reported prevalence of 68% for patient-perceived problems and 71% for listener-rated speech impairment [3]. In a study of 125 participants with PD [4], 38% placed speech among their top four concerns and, in another study, 29% of participants [5] reported speech problems to be among their greatest present difficulties. Miller et al. [6] noted how changes in communication led to increased physical and mental demands during conversation, an increased reliance on family members and/or carers, and an increased likelihood of social withdrawal. The speech of people with PD is often perceived as sounding quiet and imprecise, creating a potential social barrier to communication [7]. A qualitative study involving 24 people living with PD identified problems with speaking as an activity. The interviewees reported having to think more about speaking; weighing up the value versus the effort of speaking; having negative feelings about speaking; finding that speaking is influenced by different people and places; and having to adjust to the effect of speaking of the disease progression and their medication [8]. Overall, impairments of speech have been recognised to reduce the quality of life of people with PD [7, 9, 10].

Speech and Language Therapy (SLT) for speech difficulties in people with PD in the UK aims to improve communication. Some therapy approaches engage the person with PD in exercises to improve motor skills, others support the communication partnership between the person with PD and their communication partner, while others consider augmentation, or alternative means of communication. Following assessment and discussion with the person with PD, an individually tailored therapy plan is developed to address their needs and those of their family. For the purposes of the PD COMM trial, any of the above will be included in the intervention arm called ‘standard National Health Service (NHS) SLT’. This ‘standard NHS SLT’ is distinct from the other trial intervention arm which comprises Lee Silverman Voice Treatment (LSVT LOUD®) [11]. Within the PD COMM trial two types of SLT will be assessed: standard NHS SLT and LSVT LOUD® [11] against a no-SLT control.

Standard NHS SLT can include any of the above with the therapist selecting from multifaceted therapy components and tailoring these to the needs of the individual, their impairment and participation targets and within the constraints for that particular clinical service [6]. Typically, weekly sessions over 6–8 weeks are prescribed, either individually or in groups [12, 13]. LSVT LOUD® differs from standard NHS SLT in that it is a more prescriptive therapy. The focus of LSVT® is to ‘think loud’; improving phonation and vocal loudness through better vocal fold adduction [14] over an intervention lasting for 16 sessions. The single focus and intensive delivery is used to ‘recalibrate’ the patient, so that they recognise that a voice which sounds too loud to them is necessary for them to be understood by other people. LSVT LOUD® is increasingly being used in the NHS, but in the context of PD COMM, standard NHS SLT excludes the delivery of LSVT®.

A Cochrane review of SLT versus no intervention (last search date 11 April 2011) identified three randomised controlled trials (RCTs) of differing SLT interventions versus no intervention (*n* = 63) [15]. The SLT evaluated varied from: 10 h over 4 weeks of individual therapy focussing on prosodic features of pitch and volume reinforced by visual feedback [16]; 16 h over 4 weeks of LSVT [11]; and 35–40 h over 2 weeks improving loudness and pitch variation, respiration, voice production and intelligibility reinforced by visual feedback primarily in a group setting [17]. The review authors concluded that while outcome measures improved following SLT, the small participant numbers, low RCT quality and the possibility of publication bias, meant that the efficacy of SLT for progressive dysarthria in PD against a placebo or no intervention could not be confirmed or refuted. In another Cochrane review of six trials comparing different theoretical approaches to SLT provision for people with dysarthria (*n* = 159) [18]. All six trials assessed intelligibility and almost all of the results were not statistically significant. The exception to this was for one of the three types of perceptual ratings of speech recordings made in the study by Halpern et al. [19] for which LSVT gave the greater improvement (*n* = 14). Herd et al. [18] concluded that the small number of participants examined, the low methodological quality of the trials evaluated, and the possibility of publication bias resulted in an inability to determine the superiority of any one type of SLT over another. Since these reviews were published, two further RCTs have been reported: the PD COMM Pilot trial [13] (described below) and a trial comparing LSVT LOUD® with LSVT ARTIC® (a second type of LSVT focussing on increased movement amplitude directed predominately to the orofacial-articulatory system, but with the same dosing schedule and intensity) with a no SLT intervention control in people with PD and healthy controls [20]. The latter trial included 64 people with PD and measured sound pressure level (SPL) differences and a modified Communications Effectiveness Index (CETI-M) at 1 and 7 months. LSVT LOUD® significantly increased SPL at 1 and 7 months compared to LSVT ARTIC® and control. Both LSVT groups also showed a greater improvement than control in CETI-M at 1 month, but this difference was not maintained at 7 months. A pilot trial (PD COMM Pilot) randomised 89 people with speech problems into a three-arm, assessor-blinded RCT to assess the feasibility and to inform the trial design of a full-scale RCT [15]. Participants were randomised to either LSVT LOUD®, standard NHS SLT or no intervention control in a 1:1:1 ratio. The pilot trial showed a trend towards improvement in the LSVT LOUD® and SLT groups over the control group at 3 months, but the findings were not powered to give a definitive answer.

The PD COMM trial design was informed by our PD COMM Pilot trial, which was funded by The Dunhill Medical Trust. The PD COMM trial is funded by the UK National Institute for Health Research Health Technology Assessment programme (10/135/02). Further support was provided by Professor Lori Ramig in the form of free LSVT LOUD® training. The protocol is reported using the Standard Protocol Items: Recommendations for Interventional Trials (SPIRIT) [21] and Template for Intervention Description and Replication (TIDieR) [22] guidelines.

## Methods/design

The PD COMM trial protocol can be found on: https://www.birmingham.ac.uk/research/activity/mds/trials/bctu/trials/pd/PD-COMM/investigators/documentation.aspx (last accessed 10 September 2019). The trial received ethical approval on 7 December 2015 by the West Midlands NHS Research Ethics Committee (REC) (15/WM/0443), with protocol version 4.0 (14 November 2018) currently in effect (see the ‘Protocol amendments’ section below for details). The project is sponsored by the University of Birmingham (Research Governance Team, University of Birmingham, Birmingham, B15 2TT).

PD COMM is a multicentre, phase III, three-arm, unblinded RCT where participants are randomised in a 1:1:1 ratio to a no-intervention control group, NHS SLT or LSVT LOUD®.

The primary objective of the trial is to evaluate the clinical and cost-effectiveness of the two types of SLT versus no SLT treatment (no SLT control) for people with PD, but the trial will also compare the two types of SLT (LSVT LOUD® versus standard NHS SLT). Therefore, there will be three comparisons within the trial:

1. LSVT LOUD® versus no SLT control
2. Standard NHS SLT versus no SLT control
3. LSVT LOUD® versus standard NHS SLT

Patient-reported measures are being used to assess the participant’s perception of how their voice impacts on daily activities and their quality of life to assess clinical effectiveness. The quality of life of carers will also be assessed, and a cost-effectiveness analysis will be performed.

### Participant characteristics

People will be eligible for recruitment into the trial if they have idiopathic PD as defined by the 1988 UK PDS Brain Bank Criteria [23]; they, or their carer, report problems with speech or voice when asked and they have no signs of dementia – typically this is determined by the person with a PD clinician as per local practice. Furthermore, they must not have evidence of laryngeal pathology including: vocal nodules, a history of vocal strain or previous laryngeal surgery as such patients may not be suitable for LSVT LOUD®; and have not received SLT for speech- or voice-related problems in the past 2 years, as there is some evidence that benefits of LSVT may persist for 24 months [14].

### Identification, recruitment and randomisation

The trial is designed to align with routine care, thus minimising the burden for people with movement difficulties. Participants will usually be identified during routine clinic appointments with their clinician or Parkinson’s specialist nurse who will inform potentially eligible patients of the trial and provide a copy of the participant information sheet (PIS). The patient will be given time to review the PIS and/or go through it with a member of the team, typically the research nurse, and will be given the opportunity to ask questions. Given the low-risk nature of the trial, and the mobility limitations of the population, participants may join the trial on the same day that they discuss the trial and receive the PIS or may come back at a later date if they prefer.

Following informed consent and completion of the baseline assessment and questionnaires, the participant can be randomised into the trial. Prior to randomisation, the team will check the availability of speech and language therapists at that site. Randomisation may be deferred if the SLT intervention cannot be initiated within the set time frames – however, the participant’s baseline questionnaire needs to be completed within 2 weeks prior to randomisation, so this should be factored in to any planned delay of a patient’s randomisation. Typically the research nurse, will obtain informed consent and randomise the patient into the trial and liaise with the speech and language therapist to ensure that SLT (should they be randomised to therapy) starts within the required time frame.

Following informed consent and completion of all baseline data collection, participants will be randomised at the level of the individual via a central secure web-based randomisation system at the Birmingham Clinical Trials Unit (BCTU) to ensure concealment of the next treatment allocation. Typically, randomisation will be performed by the research nurse. The randomisation process will use a minimisation procedure with a random element. The following minimisation variables will be used:

- Age (≤ 59, 60–70, > 70 years)
- Disease severity measured using the Hoehn and Yahr [24] staging (1.0–2.5, 3.0–5.0) and
- Severity of speech measured using the Voice Handicap Index (VHI) [25] total score (≤ 33, mild 34–44, moderate 45–61, severe > 61)

Once randomised into the trial, NHS SLT should start within 4 weeks and LSVT LOUD® should start within 7 weeks to enable the intervention to be completed prior to the primary end point (at 3 months post randomisation). The trial will last for 77 months. Recruitment will take place in approximately 42 sites around the UK (see Table 1).

**Table 1:** Participating sites and principal investigators (PIs)

Trust	PI
Aneurin Bevan University Health Board	Alistair Church
Basildon and Thurrock University Hospitals NHS Foundation Trust	Sigurlaud Sveinbjornsdottir
Betsi Cadwaladr University Health Board	Sam Abraham
Blackpool Teaching Hospitals NHS Foundation Trust	David McGhee
Brighton and Sussex University Hospitals NHS Trust	Romi Saha
Cardiff and Vale University Health Board	Chris Thomas
Central London Community Healthcare NHS Trust	Ayano Funaki
East Cheshire NHS Trust	Monty Silverdale
Gloucestershire Hospitals NHS Foundation Trust	Sangeeta Kulkarni
Great Western Hospitals NHS Foundation Trust	Graham Lennox
Guy’s and St Thomas’ NHS Foundation Trust	Thomasin Andrews
Harrogate and District NHS Foundation Trust	Rosaria Buccoliero
Heart of England NHS Foundation Trust (now University Hospitals Birmingham NHS Foundation Trust)	Martha Pinkney
Homerton University Hospital NHS Foundation Trust	Laetitia Sautin
Hywel Dda University Health Board	Christopher James
Lancashire Teaching Hospitals NHS Trust	Tahir Majeed
Leeds Teaching Hospitals NHS Trust	Ioannis Mavroudis
London North West Healthcare NHS Trust	Judy Anne Juada
NHS Ayrshire and Arran	Andrew Watt
NHS Dumfries and Galloway	Shona Donaldson
NHS Forth Valley	Suvankar Pal
NHS Greater Glasgow and Clyde	Steven Wishart
NHS Highland/Argyll and Bute	Martin Wilson
NHS Lanarkshire	Helen Morgan
NHS Lothian	Gordon Duncan
NHS Tayside	Derek Sutherland
Norfolk and Norwich University Hospitals NHS Trust	Stephanie Cooper
Norfolk Community Health and Care NHS Trust	Lauren Issacs
North Bristol NHS Trust	Alan Whone
Northern Lincolnshire and Goole NHS Foundation Trust	Shankar Kamath
Pennine Acute Hospitals NHS Trust	Jason Raw
Powys Teaching UHB	Jane Price
Royal Devon and Exeter NHS Foundation Trust	Ray Sheridan
Sandwell and West Birmingham Hospitals NHS Trust	Carl Clarke
St Helens and Knowsley Hospitals NHS Trust	Dipen Gandecha
Taunton and Somerset NHS Foundation Trust	Simon Cooper
The Walton Centre NHS Foundation Trust	Malcolm Steiger
Torbay and South Devon NHS Foundation Trust	Rachel Nashed
University Hospitals Bristol NHS Foundation Trust	Theresa Allain
University Hospitals of South Manchester NHS Foundation Trust (now Manchester University NHS Foundation Trust)	David Ahearn
Bath and North East Somerset Community Health and Care Services NHS	Veronica Lyell
Wye Valley NHS Trust	Emma Wales

#### Interventions

##### Standard NHS SLT

Standard NHS SLT is not prescriptive and does not have standard content or dosage. It is, therefore, not possible to predict the number of sessions that will be provided. A survey of current UK SLT practice for PD [12] reported a median dose of six sessions delivered over 42 days. The PD COMM Pilot trial found the median dose to be six sessions (range 1–14) over an average of 9.6 weeks (standard deviation 6.1 weeks) [13].

Treatment is typically tailored to the individual’s level of difficulty or dysarthria severity and their interests as individual need and clinical constraints. Treatment will be individualised to suit each participant’s needs as per local practice. It may include impairment-based interventions, compensatory interventions and augmentative and alternative communication (ACC) strategies aimed at improving communication and participation. The participant’s family/carer(s) may be involved. Therefore, the NHS SLT arm will encompass any SLT interventions that are not LSVT LOUD® as per the LSVT LOUD® protocol.

Treatments targeted at impairment level may include exercises focussed on improving capacity, control and co-ordination of respiration, techniques for improving phonation intensity and co-ordination with respiration (but not LSVT LOUD®), and exercises to improve the range, strength and speed of the articulatory muscles [16, 17]. Behavioural therapy may include interventions aimed at reducing prosodic abnormality [26, 27] such as exercises targeting pitch, intonation, stress patterns and volume variation [16, 17, 26–28], and techniques to address the overall rate of speech [16, 17] including the use of therapeutic devices such as pacing boards [29, 30]. AAC strategies such as topic and alphabet supplementation through communication books and boards may be employed [31] along with AAC devices such as voice amplifiers, delayed auditory feedback systems and masking devices [32–34]. The practice of pitch-limiting voice treatment [35] may also be utilised within the standard SLT intervention.

The above methods may include techniques used in LSVT LOUD®, e.g. vocal intensity exercises, but will be distinct by the individualised treatment, other SLT strategies, lower intensity of delivery and (potentially) the use of group intervention.

Dose will be determined by the therapist reflecting participants’ individual needs, but the duration is unlikely to exceed 12 weeks of treatment. Sessions will be conducted by a suitably trained speech and language therapist or therapy assistant on a one-to-one or group basis per participant need and local practice. Sessions may take place in out-patient clinics, the participant’s home or in the community.

##### LSVT LOUD®

The LSVT LOUD® intervention is prescriptive and consists of four 50-min sessions per week delivered over 4 weeks [36]. Each session follows a similar structure: 25 min of repeated and intensive maximum-effort drills, and 25 min of high-effort speech production tasks [36]. Participants will also be set 5 to 10 min of home-based practice tasks on treatment days, and up to 30 min of home-based practice tasks on non-treatment days [37].

Content will consist of repeated repetitions of sustained ‘ah’ phonation, maximum fundamental frequency-range high- and low-pitch glides, and functional sentence repetition for the first half of each session, and exercises using speech production hierarchy that progresses throughout duration of the treatment programme (single word, phrases, sentences, paragraph reading, conversation) during the second half of the sessions [37]. Throughout all of the sessions, the focus of the intervention will be to ‘think loud’, maintaining the vocal loudness produced during vowel phonation throughout all other tasks during the treatment [36].

Sessions will be conducted by a suitably trained speech and language therapist or therapy assistant on a one-to-one basis. Sessions may take place in out-patient clinics, the participant’s home or remotely using tele-LSVT software. Once the exercises have been established, LSVT LOUD® may be delivered using the LSVT companion software for a proportion of the time, in line with local practice.

##### Control

The control group will not receive any SLT for their speech for 12 months, unless it becomes clinically necessary. At the end of 12 months, participants will be treated per local practice. If the participant needs SLT prior to 12 months, with their continued consent, they will remain in the trial and be followed up as usual and included in the trial analyses.

Participants are free to withdraw from the trial at any time or they may agree to continue in the trial but not comply with treatment. In the latter case they will continue to be followed up as per the protocol and the data will be analysed according to the group that they were randomised to (intention to treat).

#### Outcomes

Following the analysis of PD COMM Pilot, the design of the trial was refined to reduce the burden on participants and speech and language therapists by excluding the battery of vocal assessments and the Voice-related Quality of Life [38] questionnaire.

### Primary outcome

The primary outcome measure for the trial is patient reported Voice Handicap Index (VHI) [25] total score at 3 months.

There was some discussion about the choice of primary outcome, as most previous trials had used vocal loudness. The VHI was chosen given that it is patient-reported, brief, (and from the pilot trial) well-completed and, in our view, better reflects the focus of the trial. Alongside the VHI, a battery of patient-reported outcomes (PROs; see the ‘Secondary outcomes’ section) are being collected, which cover a range of other important areas. In the pilot trial, extensive vocal assessments were carried out alongside the PROs. We decided not to undertake vocal assessments within the PD COMM trial as (1). the additional time involved was prohibitive; (2). there was concern that since one of the trial interventions (LSVT LOUD®) specifically focussed on vocal loudness that the results might be skewed in favour of this intervention and (3). the focus of the trial was on the participants self-perception of functional communication rather than vocal loudness.

### Secondary outcomes

Patient reported: Subscales of the VHI [25]; Parkinson’s Disease Questionnaire-39 (PDQ-39) [39]; Questionnaire on Acquired Speech Disorders (QASD) [40]; EuroQol-5D (5-level version) [41]; ICEpop Capabilities Measure for Older Adults (ICECAP-O) [42]; resource utilisation; and adverse events (AEs).

Carer reported: Carer quality of life (Parkinson’s Disease Questionnaire–Carers) [43].

### Other data collected includes

Demographics including height (baseline only), weight and Hoehn and Yahr stage (at baseline and 12 months) [24]; PD medication; Abbreviated Mental Test [44] (intervention arms only), therapy logs including home-based therapy diaries, NHS therapy notes and a global rating scale (Transition item).

#### Adverse events

A risk assessment of the PD COMM trial has been performed with the SLT interventions considered to be of low risk. From the literature, the only reported AE associated with the interventions was a small increased risk of vocal strain or abuse; however, none were reported in the PD COMM Pilot trial. This risk will be minimised: speech and language therapists are trained to identify and rehabilitate vocal strain so, if present, the therapist will be able to identify and address it. No other risks are expected to arise from taking part in the trial. It is, therefore, reasonable to collect only targeted AEs.

For participants in either therapy arm, any vocal strain or abuse believed to be associated with treatment will be identified by the therapists at the participants’ therapy session and will be reported in the AE log.

In all trial arms, BCTU will also check the participant-reported resource usage form to ensure that no vocal strain or abuse has occurred following participants reporting out-patients appointments with ear, nose and throat (ENT) specialists. At the 12-month clinical visit, the medical professional will also check whether any AEs have occurred since entering the trial.

Serious adverse events (SAEs) are events that cause death, are life-threatening, require or extend an existing hospitalisation, result in persistent or significant disability or incapacity; or, are otherwise considered medically significant by the investigator. SAEs that are not related to vocal strain or abuse are excluded from expedited notification during the course of the trial and will be collected in the resource usage and 12-month clinical case report form (CRF).

#### Data collection

Data is collected in clinic at baseline and 12 months post randomisation at usual clinic appointments, and patient-reported outcome data is also collected at 3, 6 and 12 months post randomisation by postal questionnaires to the participant which are returned directly to BCTU. Participants who do not comply with their treatment allocation will be followed up as other participants unless they chose to withdraw from the trial. Data is reviewed on receipt, and return rates and completeness are closely monitored throughout the trial to ensure patient retention. The Trial Management Group review return and completeness rates and can determine whether to trigger further review or monitoring.

**Fig. 1:**
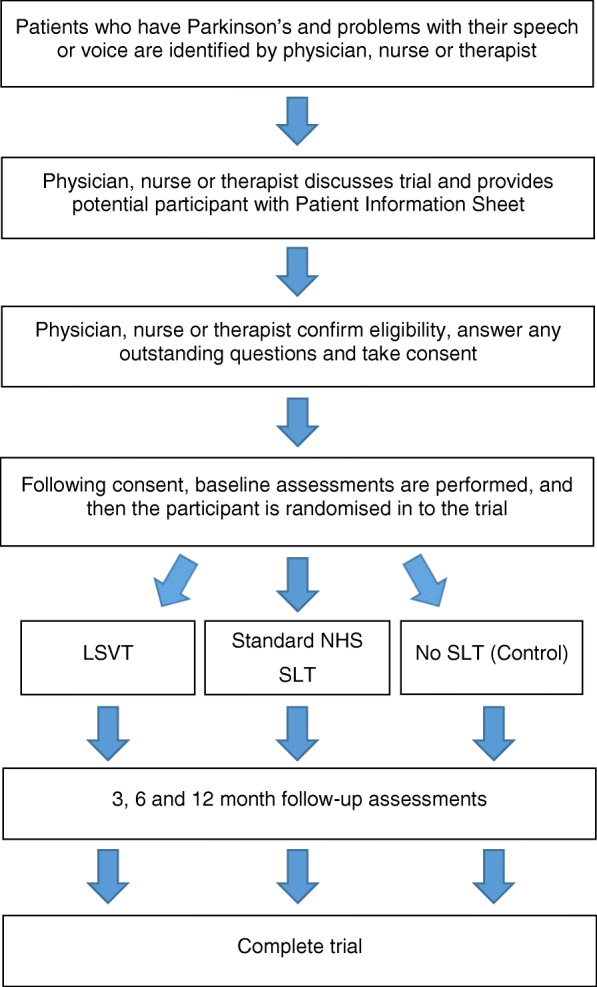
Flow diagram of trial design

Figure 1 provides a flowchart of the trial design and Table 2 denotes the schedule of events. Data is sent to BCTU where they are securely held in restricted-access areas and entered onto a bespoke trial database. For participants randomised to the intervention arms, further data around the interventions including therapy logs and therapy notes is sent to BCTU (see below). Redacted therapy notes and anonymised data sets will be sent to researchers at Glasgow Caledonian University for the intervention description analysis. This data will be integrated with the process evaluation data collected and held at the University of Bangor.

**Table 2:** Time and event schedule

Measure	Enrolment	Assessment time			
		Allocation t = 0	3 months	6 months	12 months
**Enrolment**					
Informed consent	✓				
Eligibility	✓				
Allocation		✓			
**Interventions**					
Initial interview log and treatment record form (all sessions)			✓^a^		
Home-based therapy diary			✓^b^		
**Assessments**					
Baseline case report form		✓			
VHI; PDQ-39; QASD; EQ-5D-5 L; ICECAP-O		✓	✓	✓	✓
Resource Usage Questionnaire			✓	✓	✓
Global rating score (Transition item)			✓		
PDQ-Carer		✓	✓	✓	✓
Adverse event log			✓^c^		
12-month case report form					✓

^a^Following each therapy session for participants in the two SLT treatment arms only

^b^Completed at home by the participant as recommended in their SLT therapy session

^c^Only required for participants randomised to a treatment arm

Key: *EQ-5D* EuroQol 5-dimension, 5-level questionnaire, *ICECAP-O* ICEpop Capabilities Measure for Older Adults, *QASD* Questionnaire on Acquired Speech Disorders, *PDQ-39* Parkinson’s Disease Questionnaire-39, *SLT* Speech and Language Therapy, *VHI* Voice Handicap Index

#### Treatment dose and fidelity

The speech and language therapists will complete an initial interview log including the Abbreviated Mental Test and intervention record forms at each treatment session for participants receiving SLT. Furthermore, in the LSVT LOUD® arm and where prescribed in the NHS SLT arm, participants will complete home-based therapy diaries. Therapists will also complete standard NHS therapy notes; upon trial treatment completion, a pseudo-anonymised (i.e. participant is only identified by trial number) version of these will be sent to the research team.

#### Process evaluation

In order to evaluate the implementation of PD COMM interventions, a process evaluation will be carried out alongside PD COMM. The process evaluation team led by Bangor University will employ a number of approaches to data collection, including:

- Qualitative interviews with PD COMM participants
- Qualitative interviews with PD COMM therapists
- Critical incident reports
- Therapist’s questionnaire

A detailed protocol for these analyses including intervention description has been published [45].

The analysis of process evaluation data will focus on the practical implementation of the trial interventions, including how these were tailored to individual patient and other circumstances.

#### Sample size

The primary outcome is the mean difference in the VHI total score at 3 months across the three comparisons: LSVT LOUD® versus control; standard NHS SLT versus control; and LSVT LOUD® versus standard NHS SLT. Data from the PD COMM Pilot trial was used to inform the sample size calculations for this trial as the minimal clinically important difference (MCID) for the VHI has not been established in PD patients. In the PD COMM Pilot trial, a difference of around 10 points in VHI total score was observed at 3 months between SLT and control for both types of SLT (standard NHS and LSVT LOUD®) versus control comparisons. To detect a 10-point difference in VHI total score between arms at 3 months (using a two-sided *t* test and the upper standard deviation of 26.27 obtained from the VHI baseline data from the pilot trial; effect size 0.38), with 80% power and *α* = 0.01, we need 163 participants per arm. Allowing for 10% drop-out will require 182 participants per arm, so 546 participants in total.

#### Statistical analysis

All primary analyses (for both the primary and secondary outcomes) will be by intention to treat. Participants will be analysed in the treatment group to which they were randomised, and all participants will be included whether or not they received the allocated treatment. This is to avoid any potential bias in the analysis. For all tests, summary statistics (e.g. mean differences) will be reported along with 95% confidence intervals and *p* values from two-sided tests. A *p* value of < 0.01 will be considered statistically significant, as per the sample size calculations to take into account the multiple treatment comparisons being undertaken.

There will be no interim analyses.

The analysis and interpretation of qualitative data, and its integration with quantitative data on intervention provision, will be performed between researchers at Bangor University (Professor Christopher Burton) and Glasgow Caledonian University (Professor Marian Brady), with input from University College London (Dr Christina Smith) and King’s College London (Professor Catherine Sackley).

### Primary outcome analysis

The primary outcome measure is the VHI total score at 3 months. A linear regression model will be used to estimate differences in the VHI total score at 3 months between the two arms of interest, with the VHI baseline score and the minimisation variables age and severity of PD (Hoehn and Yahr) included in the model as covariates.

### Secondary outcome analyses

The majority of the secondary outcome measures (e.g. PDQ-39) are continuous measurements and will be analysed in a similar way to that described for the primary analysis: a linear regression analysis adjusting for relevant baseline score and all of the minimisation variables (baseline VHI, age and severity of PD). As per the primary outcome, the primary analysis for the secondary outcomes will be based on the 3-month data.

To assess whether any treatment effect is maintained, participant- and carer-completed questionnaires are also being collected at 6 and 12 months post randomisation. Data collected at 6 and 12 months will be analysed using the same methods as described above. Further analysis using a repeated measures model will also be performed using all data over the 3-, 6- and 12-month assessment points.

A global rating scale (Transition item) will be completed by the participant and carer separately at the 3-month time point. This data will be used to calculate an MCID for the VHI in this population.

Adverse events and safety data will be summarised descriptively by treatment arm, and the number of events and percentage of participants experiencing any AE reported. It is not expected that there will be many AEs as a result of the intervention, but the number of participants reporting an AE will be compared using a chi-squared test, with relative risks and 95% confidence intervals reported (if appropriate).

### Planned subgroup analyses

Subgroup analyses will be performed for the primary outcome to assess whether there are differences in treatment effect by the minimisation variables: age; baseline voice severity (as measured by VHI); and PD severity (as measured by Hoehn and Yahr). The trial is not powered to detect differences in treatment effect in these subgroups and, therefore, these analyses will be treated as purely hypothesis generating.

#### Health economics analysis

The economic evaluation will estimate the cost-effectiveness of LSVT LOUD® or standard NHS SLT compared to no SLT treatment (control) in PD. The base-case economic evaluation will be undertaken from the UK NHS and personal social services (PSS) perspective, with further analysis from a broader societal perspective, over 12 months’ follow-up.

A cost-effectiveness analysis will use the primary outcome (VHI) to calculate the cost per unit improvement in VHI score, and a cost-utility analysis will use responses from the EQ-5D-5 L to calculate cost per quality-adjusted life year (QALY) gained. Resource-use data will be collected on PD-related medication, primary care and secondary care healthcare utilisation, including the use of therapy services, and use of social services including formal care. Further information will be collected on time off work, participant out-of-pocket costs and costs incurred by informal carers, in order to inform analysis from a societal perspective. The cost of delivering the LSVT LOUD® intervention and NHS SLT, including length and number of sessions and any training required will be determined within the trial. Data will be collected using a participant-completed resource utilisation questionnaire (at 3, 6 and 12 months) and the therapist-completed initial interview logs and treatment record forms. Unit costs from routine sources will be applied to resource-use data [46, 47]. Health-related quality of life will be assessed using the EQ-5D-5 L [41] collected at baseline, 3, 6 and 12 months. The crosswalk value set will be applied to patient responses to obtain utility scores, in line with current NICE recommendations and QALYs calculated using the ‘area under the curve’ approach. The ICECAP-O [42] will also be used to capture changes in participants’ capabilities, allowing a broader assessment of benefits to patients.

Incremental cost-effectiveness and cost-utility analyses will be undertaken to estimate the incremental cost per unit of outcome gained, adjusting for baseline covariates. Two sets of comparisons will be undertaken. In line with convention, strategies will be ordered from least to greatest cost, with each strategy compared against the next more costly strategy, and strategies which are dominated or extendedly dominated subsequently excluded. A separate analysis will also consider the three pair-wise comparisons as specified in the statistical analysis. Both deterministic and probability sensitivity analysis will be undertaken and cost-effectiveness acceptability curves will be produced to reflect the probability the intervention will be cost-effective at different willingness to pay thresholds, in terms of cost per unit of outcome gained.

#### Dissemination

The trial results will be disseminated widely through scientific conferences and peer-reviewed publications. Collaborators will be informed of the trial results at a national trial results meeting and participants will be sent a newsletter thanking them for their support and informing them of the trial results. We will also use social media and work with the Universities media services, National Institute of Health Research (NIHR) Health Technology Assessment (NIHR HTA) dissemination services and the Parkinson’s UK charity to broaden our dissemination to key stake-holders and the wider community.

#### Roles and responsibilities

The study design, collection, management, analysis and interpretation of the data, publishing the data is the responsibility of the chief investigator, and the Collaborative Group, the trial is managed by the BCTU, with additional support for Scottish sites being provided by Glasgow Caledonian University. The process evaluation is managed by the University of Bangor and the intervention description analysis is managed by Glasgow Caledonian University. The sponsor and funder do not have a role in the above activities.

Oversight of the trial is performed by the Trial Management Group, an independent Data Monitoring Committee (DMC) and Trial Steering Committee (TSC). The DMC and TSC membership (see Table 3) was agreed with the funder; these committees meet at least annually to review the data and progress of the trial. Annual reports are submitted to the NHS REC and monthly updates against pre-determined milestones are sent to the funder.

**Table 3:** Trial Oversight Committees

**Trial Management Group**	**Data Monitoring Committee**
Professor Catherine Sackley (chief investigator)	Dr Carl Counsell (chair), Consultant Neurologist, University of Aberdeen
Ms Pui Au	
Ms Gillian Beeston	Dr Katherine Deane, Senior Lecturer, University of East Anglia
Professor Marian Brady	
Professor Christopher Burton	Dr Louise Hiller, Statistician, University of Warwick
Ms Maria Caulfield	
Professor Carl E Clarke	**Trial Steering Committee**
Ms Sylvia Dickson	Dr Lisa Shaw (chair), Senior Research Associate, University of Newcastle upon Tyne
Dr Sue Jowett	
Dr Patricia Masterson Algar	Mr Chris Jeffery, Patient and Public Involvement Representative
Dr Avril Nicoll	
Mrs Smitaa Patel	Ms Michelle Collinson, Senior Medical Statistician, University of Leeds
Dr Cally Rick	
Mrs Natalie Rowland	Dr Linsay Pennington, Senior Lecturer and Speech and Language Therapist, University of Newcastle upon Tyne
Dr Christina Smith	
Ms Rebecca Woolley	Professor Catherine Sackley, Professor of Rehabilitation, King’s College London
**Former members**	Dr Paul Worth, Consultant in Neurology, Cambridge University Hospitals NHS Foundation Trust
Mr Francis Dowling	
Mr Max Hughes	
	**Former member:**
	Mr John Carrington, Patient and Public Involvement Representative
	Professor Adam Gordon, Professor in Medicine of Older People, University of Nottingham
	Dr Simon Horton, Lecturer and Speech and Language Therapist, University of East Anglia

## Discussion

The PD COMM trial is already the largest trial of SLT in PD to date (as of 10 September 2019, 329 participants have been recruited). It will provide robust evidence as to the effectiveness and cost-effectiveness of two types of SLT for people with PD enabling them, their clinicians and NHS decisions-makers to make informed choices.

There will also be benefits beyond the immediate trial results: the group has developed a research network of speech and language therapists in 42 sites around the UK, participated, and provided training in, LSVT LOUD® (courtesy of Dr Lori Ramig), trials, other aspects of PD and research into SLT and information on best practice. This project will lay the foundation for further trials in SLT and lower the barriers to future research. Furthermore, we will develop a clear understanding of the range of SLT practice around the UK for people with speech or voice problems as a consequence of PD, in terms of dosage, content and availability.

## Trial status

Protocol version number and date: version 4.0 (14 November 2018).

The first participant was recruited into the trial on 11 October 2016. Recruitment is ongoing with an expected recruitment end date of 30 November 2020.

## Data Availability

Following the publication of the main trial results anonymous data sets will be available on an individual case-by-case basis in accordance with the University of Birmingham, Birmingham Clinical Trials Unit’s Standard Operating Procedures with agreement from Professor Catherine Sackley and King’s College, London.

## Abbreviations

AE: Adverse event
ACC: Augmentative and alternative communication
BCTU: Birmingham Clinical Trials Unit
ENT: Ear, nose and throat
ICECAP-O: ICEpop Capabilities Measure for Older Adults
ISRCTN: International Standard Randomised Controlled Trials Number
LSVT LOUD®: Lee Silverman Voice Treatment
MCID: Minimal clinically important difference
CETI-M: Modified Communications Effectiveness Index
PD: Parkinson’s disease
PDQ-39: Parkinson’s Disease Questionnaire-39
PIS: Patient information sheet
RCTs: Randomised controlled trials
SAE: Serious adverse event
SPL: Sound pressure level
SLT: Speech and Language Therapy
VRQOL: Voice-related Quality of Life
VHI: Voice Handicap Index
